# Applications of Clustered Regularly Interspaced Short Palindromic Repeats (CRISPR) as a Genetic Scalpel for the Treatment of Cancer: A Translational Narrative Review

**DOI:** 10.7759/cureus.50031

**Published:** 2023-12-06

**Authors:** Riddhi Mondal, Niki Brahmbhatt, Sahibjot K Sandhu, Hetvi Shah, Mandeepsinh Vashi, Siddharth Kamal Gandhi, Priyansh Patel

**Affiliations:** 1 Department of Internal Medicine, Jagannath Gupta Institute of Medical Sciences and Hospital, Kolkata, IND; 2 Department of Internal Medicine, OneStepForward Research Initiative, Ahmedabad, IND; 3 Department of Internal Medicine, Gujarat Medical Education & Research Society (GMERS) Medical College Sola, Ahmedabad, IND; 4 Department of Internal Medicine, Sri Guru Ram Das Institute of Medical Sciences and Research, Amritsar, IND; 5 Department of Anesthesia, Dr L H Hiranandani Hospital, Mumbai, IND; 6 Department of Internal Medicine, Surat Municipal Institute of Medical Education and Research, Surat, IND; 7 Department of Internal Medicine, Shri M. P. Shah Government Medical College, Jamnagar, IND; 8 Department of Internal Medicine, Medical College Baroda, Vadodara, IND

**Keywords:** genetics, treatment of cancer, crispr (clustered regularly interspaced short palindromic repeats)/cas (crispr-associated protein), crispr-cas system (clustered regularly interspaced short palindromic repeats)-crispr associated system, crispr

## Abstract

Cancer remains a global health challenge with high prevalence and mortality rates, imposing significant financial and emotional burdens on affected families. However, hope lies in genetic manipulation, with a focus on innovative approaches to combat genetically linked cancers. Clustered regularly interspaced short palindromic repeats (CRISPR), an adaptive immune system found in various bacteria and archaea, hold immense potential. We searched articles on PubMed Central, Medline, and PubMed databases indexed journals. The keywords from the research topic, i.e., "CRISPR AND genetic therapy," were searched, and we found 3397 articles. Following this, we explored the medical subject headings (MeSH) glossary and created a search strategy "Clustered Regularly Interspaced Short Palindromic Repeats"[Mesh] AND "Genetic Therapy"[Majr] and after applying a variety of filters we included 30 studies in our review. CRISPR consists of unique spacers and CRISPR-associated (Cas) genes, operating through acquisition, CRISPR ribonucleic acid (crRNA) biogenesis, and target interference phases. The type II CRISPR-Cas9 system is a well-researched avenue for gene editing, with Cas9 cleaving specific genomic regions and initiating deoxyribonucleic acid (DNA) repair mechanisms. Cancer results from genetic alterations, leading to tumor development with properties like metastasis. CRISPR/Cas9 offers precise genome editing to inhibit tumor formation by removing specific genomic sequences and promoting DNA repair. Challenges in CRISPR's use for cancer therapy, including delivery methods, cell adaptation, and ethical concerns, are recognized. Beyond cancer, CRISPR finds diverse applications in infectious diseases and non-cancerous conditions, signifying its transformative potential in modern medicine. CRISPR technology represents a groundbreaking frontier in cancer therapy and beyond, offering insights into genetic editing, disease mechanisms, and potential cures. Despite challenges and ethical considerations, precise genome editing promises improved cancer treatments and innovative medical interventions in the future.

## Introduction and background

In 2023, statistical calculations by the American Cancer Society (ACS) show that around two million new cancer cases are expected to occur in the United States (US) alone, with more than half a million projected cancer deaths [1]. Because of the high rate of occurrence as well as the considerable mortality, cancer is a curse that has remained the second-biggest cause of death in the whole world since 2019 [2]. Added to that are the financial repercussions a family must face when the patient diagnosed with cancer requires several rounds of chemotherapy and extensive surgeries with high risks of fatality. However, current research is being focused on devising methods using genetic manipulation to cure malignancies related to genetic mutation. The clustered regularly interspaced short palindromic repeats (CRISPR) is a breakthrough discovery in the field of modern science. Some eubacteria and all archaebacteria have already displayed the presence of the CRISPR/CRISPR-associated (Cas) nuclease system in them [3]. It acts as an acquired immunity by allowing the organisms to protect themselves against invading viruses and plasmids. The CRISPR array has short variable deoxyribonucleic acid (DNA) sequences that make up unique spacers. And these are accompanied by the Cas gene. The Cas gene can encode the Cas protein [4]. The CRISPR/Cas immune system acts in three phases for neutralizing the target pathogen, namely, acquisition, CRISPR ribonucleic acid (crRNA) biogenesis, and target interference [5].

There are two classes of the CRISPR/Cas system. Among the CRISPR/Cas subdivisions, the type II CRISPR/Cas9 system is the most researched avenue for gene editing [6]. Cas9 protein has a giant structure with HNH nuclease and RuvC-like nuclease domains [7]. These can cleave the target DNA and result in double-stranded DNA breakings (DSBs) after targeting the specific regions of the genome [8]. Then, each cleaved end will be repaired by DNA repair methods like homology-directed repair (HDR), classical non-homologous end joining (NHEJ), and micro-homology-mediated end joining (mHMEJ). These repair methods can be employed to treat and ultimately cure diseases [8]. Cancer is caused by changes at the genetic level in the cells which later become tumor cells and can acquire properties like metastasis and lack of contact inhibition [9]. CRISPR/Cas9 is now being used to cut out the specific genomic codes using nuclease and cause DNA repair to edit the genomes and inhibit tumor formation [10]. Yet, the use of CRISPR also introduces numerous practical and technical hurdles, primarily related to delivery methods, management of repair mechanisms, unintended effects both at the target site and elsewhere, as well as contentious ethical concerns [10]. In this narrative review, we provide an update on the literature that deals with the safety and efficacy of CRISPR as a genetic scalpel to cleave out the code that causes cancer.

Methodology

Working in collaboration with other authors of this paper, we searched articles on PubMed Central, Medline, and PubMed databases indexed journals. The keywords from the research topic, i.e., "CRISPR AND genetic therapy," were searched, and we found 3397 articles. Following this, we explored the medical subject headings (MeSH) glossary and created a search strategy "Clustered Regularly Interspaced Short Palindromic Repeats"[Mesh] AND "Genetic Therapy"[Majr] and were left with 187 articles. No time parameters were set. Moreover, publications that were not in the English language were excluded. Articles were excluded where the free full text could not be retrieved. Any scholarly articles and comprehensive studies that did not maintain a direct correlation with the Human species were expeditiously excised from the collection, which left us with 44 results in the end. All articles underwent meticulous scrutiny, and any divergent perspectives were deliberated upon until a consensus was achieved. Following thorough discourse among all contributing authors, a unanimous decision was made to incorporate a total of 30 studies into the comprehensive review.

## Review

Mechanism of CRISPR

**Figure 1:**
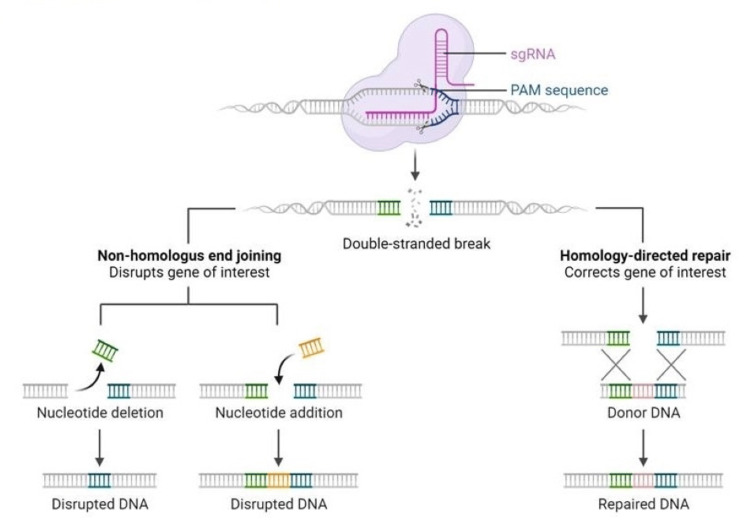
Mechanism of CRISPR/Cas9 gene editing DNA: Deoxyribonucleic acid, sgRNA: Single guide RNA, PAM: Proto spacer adjacent motif, CRISPR: Clustered regularly interspaced short palindromic repeats, Cas: CRISPR-associated Adapted from "CRISPR/Cas9 gene editing" by BioRender.com (2023)

CRISPR/Cas systems are primarily divided into two classes, class I and class II. In class I CRISPR/Cas systems, multiple Cas proteins are required for their functionality [11]. In contrast, class II systems, which are more popular for gene editing, only require a single protein [11]. The type II CRISPR/Cas9 system consists of the Cas9 protein, crRNA, and trans-activating RNA (tracrRNA) [12]. Cas9 endonuclease can form a complex with either crRNA or tracrRNA [13]. Through engineered design, crRNA and tracrRNA can be combined to form guide RNA (gRNA) with a guiding capability [12]. This gRNA can direct Cas9 to precisely cut DNA, enabling gene knockout and insertion [12]. A complex made up of Cas9 and gRNA, associates with the viral target DNA in the spacer region using the proto-spacer adjacent motif (PAM) sequence [14]. When Cas9 identifies the target DNA sequence by recognizing crRNA, it produces a DSB just before the PAM region, resulting in blunt ends [14]. These DSBs can be repaired by NHEJ (via knocking out of genes) or HDR (knocking in of genes), where deletion or insertion of several base pairs take place [15]. HDR is a precise editing method using a DNA repair template, allowing exogenous sequence insertion [13]. Conversely, the NHEJ pathway, being error-prone without needing a template, often creates insertion-deletion (indel) mutations, resulting in gene knockout [13]. The donor DNA with the desired sequence is incorporated with the parent one at the specific site and new DNA strands are synthesized removing the Cas protein at the end of the whole process [14]. The advent of CRISPR/Cas gene editing technology opens up new horizons for editing human genes [12]. We foresee a transformative period in cancer biology where CRISPR-driven genome engineering facilitates a thorough analysis of oncogenic signaling pathways through gene editing [15]. The mechanism of CRISPR/Cas9 gene editing is shown in Figure 1.

Applications of CRISPR

The invention of CRISPR/Cas9 has revolutionized molecular biology, and its uses have been extended to several fields with the help of research. From the point of view of medicine, CRISPR-based technologies are now virtually indispensable [16]. Here are the multifarious uses of CRISPR/Cas9,

Use of CRISPR in Hepatocellular Carcinoma

With the help of knowledge regarding the nuclear receptor binding set domain-containing protein 1 (NSD1), Zhang et al. (2019) have found that its somatic dysregulation is associated with hepatocellular carcinoma [17]. They performed quantitative real-time polymerase chain reaction (RT-qPCR) and western blot analysis to pinpoint the expression of NSD1 in tissue affected in hepatocellular carcinoma. They also constructed an NSD1 knockout cell line which yielded results that showed inhibition of proliferation and migration of hepatocellular carcinoma cells [17].

Use of CRISPR in Breast Carcinoma

MicroRNA-3662 (miR-3622) is expressed to some extent in all the cells of the human body. But their expression is more in cancer cells, and even more in cells of triple-negative breast cancer [18]. In an original study by Yi et al. (2022), they outlined how the CRISPR/Cas9-mediated inhibition or knockout of endogenous, mature miR-3662 in triple-negative breast carcinoma cells suppresses their proliferation and migration [18]. This, in turn, decreases tumorigenesis [18].

Use of CRISPR in Lung Carcinoma

Through somatic editing, CRISPR has successfully modeled lung cancer in vivo. Notably, CRISPR constructs supplied through viruses or DNA transfection are generally accessible to lung tissue [19]. Platt et al. (2014) created CRE-dependent Cas9 knock-in mice and delivered an adenovirus vector harboring single gRNA (sgRNA) targeting TP53, LKB1, and KRAS, as well as a structure containing a KrasG12D HDR donor [19]. In less than two months, this delivery caused the formation of multiple tumors, spanning from alveolar carcinomas to invasive adenocarcinoma [19]. To model lung cancer disease, administered lentiviruses containing sgRNA against PTEN, NPX2-1, and APC as well as Cas9 and CRE intra-tracheally into LoxP KrasG12D positive and p53flox/flox transgenic mice [20]. For the study of small-cell lung cancer (SCLC), Sánchez-Rivera et al. (2014) injected into mice transgenic for p53flox/flox and Rb1flox/flox two constructs, one containing Cas9 and Csy4 (an RNA endonuclease) and another carrying sgRNA against p107 and p130 and CRE [20]. In addition, Wu et al. (2020) suggested that this novel approach could be feasible and safe in targeting genes that suppress tumors in SCLC, which could pave the way for creating an original potential therapy toward the progression of lung carcinomas [21]. Cheung et al. (2018) used CRISPR/Cas9 technology for genome editing to aim for the point mutation of endothelial growth factor receptor (EGFR) in lung tumors [22].

Use of CRISPR in Colorectal Carcinoma

Li et al. (2017) showed that lentiviral transmission of Cas9/sgRNA into the CaCO-2 cell types knocked off the Par3L protein in colorectal tumor cells by decreasing cell growth, causing death, and stimulating signaling cascade-3 [23]. They found that Par3L peptides are more responsive to anti-tumor chemotherapy treatments and hypothesized that CRISPR/Cas9 could reduce adenosine monophosphate-activated protein kinase (AMPK) signaling transduction to preserve colon cancer cell survival by inactivating Par3L [23].

Use of CRISPR in Prostatic Carcinoma

Evidence revealed that in vitro lentiviral delivery of Cas9/sgRNA affects the androgen receptor (AR) in a prostate tumor cell type [24]. They found that CRISPR/Cas9-AR silencing reduced prostate tumor cell growth by reducing cell proliferation and increasing apoptosis. Thus, CRISPR/Cas9 may revolutionize prostate tumor treatment in the future [24].

Use of CRISPR in Reducing Carcinogenic Viruses

In 2019, a team of researchers began researching the benefits and safety of CRISPR/Cas9-HPV E6/E7 and TALEN-HPV E6/E7 for treating human papillomavirus (HPV) persistent and HPV-related cervical intraepithelial neoplasia [25]. Epstein-Barr virus (EBV), a herpes virus, is linked to Burkitt's lymphoma, Hodgkin's disease, nasopharyngeal carcinoma, and gastric cancer. CRISPR/Cas9 was found to be a viable method for reducing EBV infection by deleting EBV genes [26].

Miscellaneous Uses of CRISPR

While the focus of our review stays on the use of CRISPR in cancer therapy, it is imperative that its other uses are also elucidated while speaking of the various applications of CRISPR.

The role of CRISPR in the diagnosis of non-cancerous diseases and treatment of various genetic conditions: STOPCovidv2, a CRISPR-based detection solution for SARS-CoV-2, has evidently simplified COVID-19 diagnosis with superior sensitivity and specificity compared to the RT-PCR [16]. Food and Drug Administration (FDA) approval has also highlighted its significance in combating the pandemic. Moreover, transthyretin (TTR) amyloidosis, an autosomal disorder caused by TTR gene mutations, has seen promising results in therapy using CRISPR-based gene editing with NTLA-2001 [16]. FDA has approved this method as an orphan drug due to its effectiveness in reducing TTR levels in patients [27]. Also, CRISPR/Cas9 was utilized to modify the BCL11A enhancer sequence in hematopoietic stem cells (HSCs), leading to reduced BCL11A enhancer expression and increased production of fetal hemoglobin and gamma-globin, benefiting sickle cell disease and transfusion-dependent beta-thalassemia patients [27].

Understanding diseases at the epigenetics level with CRISPR: CRISPR/Cas9's dCas9-KRAB can elucidate epigenetic mechanisms in gene regulation by targeting enhancers like HS2 [28]. This binding renders chromatin at both enhancer and promoter sites inaccessible, effectively silencing genes. This precision aids in unraveling genetic regulation mechanisms [28].

Understanding of infectious diseases with CRISPR: CRISPR/Cas9 has leveraged spacer acquisition to retain short fragments of genetic material from pathogens like phages or plasmids [29]. Upon re-infection, it identifies and targets pathogen DNA using these spacers. Researchers have harnessed this natural mechanism for precise genetic material targeting and studying disease processes [29].

Challenges in effectively implementing CRISPR

With the recent progress in research regarding genome editing based on the CRISPR/Cas9 system, some concerns have come up [30,31]. Firstly, any medical advancement is often scrutinized for possible side effects, and CRISPR is no exception. The most commonly used method for ex-vivo Cas9 nuclease delivery, electroporation, can cause cell death [30,32]. Also, some lipid-based transfection methods use commercial delivery kits, such as the lipofectamin family of reagents for Cas9 delivery [33,34]. But even then, toxicity issues and cell morphological changes resulting from transfection remain a cause of concern [35]. Secondly, if the edited cells adapt, we will see that the revised protein product is produced in the quantities to show its therapeutic effect. Whereas if the edited cells do not survive, the therapeutic effect cannot be expected [6]. Thirdly, the conventional ex-vivo delivery of Cas9 nuclease and its sgRNAs was the first step toward immunotherapy with CRISPR [36]. It was done using plasmids (independently replicating circular DNA of the eubacteria) and some classical viruses like Adenovirus, Lentivirus, Retrovirus, and Adeno-associated viruses [15]. As reported by Yip et al., electroporation is the most commonly used method for ex-vivo delivery of CRISPR, which heavily relies on the electrical properties that vary from cell to cell [37]. Thus, not all the cells will be receptive when ex-vivo delivery by electroporation is attempted. Considering these aspects, Afolabi et al. suggested that in vivo delivery methods provide more significant therapeutic advantages [38]. However, they also indicated that in vivo delivery of Cas9 nuclease shows a lack of specificity [38]. Thus, more studies are needed to delve into CRISPR/Cas9 employment methods, which will be specific and more accurate.

Limitation

It is imperative to acknowledge the existence of certain constraints within this study. The body of research we examined exclusively comprised works sourced from books, documents, clinical trials, meta-analyses, randomized controlled trials, and traditional and systematic reviews, all without any temporal restrictions on the publication dates. As a result, our selection criteria encompassed only freely accessible full-text articles, potentially omitting pertinent data. Notably, we did not incorporate specific gender or age parameters. Additionally, it's crucial to note that our review was restricted to papers published exclusively in English, thereby excluding studies available in other languages. There are some ethical issues with regards to CRISPR that might pose as limitations to studies involving its use in cancer therapy. After starting in 2012, genetic mutation by CRISPR/Cas9 took over the world of molecular biology. However, the application of CRISPR in human germ-line editing has a moderate risk level, according to a study by Shinwari et al [39]. They have also mentioned that CRISPR can easily be used for genetic enhancement where people can apply it to choose the traits they want [40]. However, genetic enhancement is now banned in most countries except for the United Kingdom (UK). Also, many economic interests revolve around CRISPR. Owing to these ethical reasons, research on CRISPR has become increasingly difficult [40].

## Conclusions

In this review, we described in detail the utilization of CRISPR/Cas9 technology in cancer therapy and various other diseases in depth. We discuss the mechanism and target actions of CRISPR on the genetic level while also describing its applications in multiple types of cancers like liver, breast, and lung in detail. We combined and reviewed numerous clinical studies for CRISPR use in the treatment of oncogenic virus-related cancers. Furthermore, the role of CRISPR technology was well established in diagnosing non-cancerous diseases and their treatment. There have been successful results of CRISPR use for diagnosing SARS-CoV 2 and in patients with transfusion-dependent hemoglobinopathies like sickle cell anemia and thalassemia. This article can also benefit biomolecular engineering using CRISPR technology in primary health sciences. However, in addition to the ethical concerns of using CRISPR in human trials, there are some challenges related to it involving lack of specificity in vivo delivery and side effects of cell toxicity. However, the available evidence for most of these challenges is limited, highlighting the necessity for additional research trials in the future to determine the effectiveness of CRISPR in molecular biology.
